# Effect of the G-Protein-Coupled Receptor T2R14 on Proliferation and Cell Population Growth in Oral Cancer Cells

**DOI:** 10.3390/cells15030279

**Published:** 2026-02-01

**Authors:** Yongqiang Chen, Manikanta Kella, Kayla Austin, Rajinder P. Bhullar, Prashen Chelikani

**Affiliations:** Manitoba Chemosensory Biology Research Group, Department of Oral Biology, Dr. Gerald Niznick College of Dentistry, University of Manitoba, Winnipeg, MB R3E 0W2, Canada; yongqiang.chen@umanitoba.ca (Y.C.); kellvnsm@myumanitoba.ca (M.K.); austink1@myumanitoba.ca (K.A.)

**Keywords:** colony formation, cell population growth, cell viability, G-protein-coupled receptor, oral cancer, oral squamous cell carcinoma, proliferating cell nuclear antigen, taste receptor, taste receptor type 2 member 14

## Abstract

**Highlights:**

**What are the main findings?**

- T2R14 knockout increased proliferation and cell population growth in oral cancer cells, suggesting that T2R14 can act as a tumor suppressor.
- External database analysis suggests that a higher TAS2R14 mRNA level is associated with a higher overall survival probability in patients with oral cancer.

**What are the implications of the main findings?**

- The study’s findings highlight a research approach by measuring cell population growth in cancer biology.
- The study’s findings inform the development of new strategies to advance therapies for oral cancer and possibly other cancers by targeting T2R14, such as achieving a high intracellular T2R14 level in cancer cells.

**Abstract:**

Oral cancer is a leading cause of cancer-related deaths and significantly affects the quality of life of patients. However, many of its mechanisms remain unclear, and its treatment needs improvement. The G-protein-coupled receptor taste receptor type 2 member 14 (T2R14 or TAS2R14) is expressed in various cancer types. However, few studies have investigated its roles in oral cancer, and its effects on oral cancer cell proliferation and growth are unknown. This study aimed to examine T2R14’s impact on proliferation and cell population growth (CPG) of oral cancer cells. TAS2R14 gene knockout was performed, and cell numbers, cell viability, and colony formation were measured. This study showed that TAS2R14 knockout in oral cancer cells significantly decreased calcium mobilization, increased cell numbers, colony formation, the proliferation marker proliferating cell nuclear antigen, and the phosphorylation of mechanistic target of rapamycin, but did not affect cell viability. These observations are consistent with the clinical data that higher TAS2R14 mRNA expression is associated with better survival of patients with oral cancer. Therefore, T2R14 downregulation increased oral cancer CPG, suggesting a tumor-suppressor-like role. The study’s findings could improve our understanding of T2R14 mechanisms and help develop strategies to advance oral cancer treatment by targeting T2R14.

## 1. Introduction

Oral cancer is the primary subtype of head and neck cancers; it initiates in the oral cavity and oropharyngeal regions, with oral squamous cell carcinoma (OSCC) accounting for over 90% of cases [1,2]. According to the Global Cancer Observatory, the incidence and mortality of cancers of the lip and oral cavity ranked 16th and 15th worldwide in 2022, respectively, with 389,846 cases and 188,438 deaths [3]. Oral cancer development includes the following stages: Stage 0: in situ carcinoma formation; Stage 1: formation of a small tumor confined to its original organ; Stages 2 and 3: formation of a large tumor that grows outside the original organ in nearby tissues; and Stage 4: spread of cancer cells to distant places of the body via the blood and/or the lymphatic system, leading to formation of secondary tumors (metastasis) [1]. It can be affected by many factors, such as oral potentially malignant disorders [4], the consumption of tobacco and alcohol [2,5], diet [2,6], microbiome [2,7], human papilloma virus (HPV) infections [1,8], oral bacteria-mediated autophagy [9], and non-coding RNAs [10]. Effective treatment of oral cancer requires a comprehensive understanding of its epidemiology, diagnosis, and progression [11], and therapeutic resistance is the biggest obstacle to treatment. Progression mechanisms and therapeutic resistance of oral cancer are influenced by genetic factors and signaling pathways, including p53, p63, p73, HPV infections, retinoblastoma, p16, epidermal growth factor receptor, rat sarcoma virus, aurora kinases A and B, Notch, the phosphatase and tensin homolog/mechanistic target of rapamycin (mTOR)/AKT/phosphoinositide 3-kinase pathway, cellular mesenchymal–epithelial transition factor, the janus kinases/signal transducer and activator of transcription pathway, the mitogen-activated protein kinase pathway, the Fas/Fas ligand/matrix metalloproteinase pathway, FAT atypical cadherin 1, caspase-8, the DNA damage repair system, epigenetic changes, microRNAs, as well as cancer stem cells [1]. However, the mechanisms of oral cancer progression are still not fully understood, and its diagnosis and treatment need to be improved.

G-protein-coupled receptors (GPCRs) are a large and diverse family of plasma membrane proteins that transmit signals from the outside of the cell into the inside, playing a role in various diseases, including cancer. Taste receptor type 2 member 14 (also called bitter taste receptor 14 or T2R14 or TAS2R14) is a member of the bitter taste receptor family, which is a subfamily of GPCRs and provokes signaling in response to tastant stimulation; among bitter taste receptors, it is the most promiscuous, responding to the largest number of agonists [12]. Recent studies have investigated the roles of T2R14 in gingival cells, focusing on autophagy [13], and in head and neck squamous cell carcinoma (HNSCC) cells, focusing on apoptotic cell death [14]. However, the role(s) of T2R14 in the progression of oral cancer remain largely unknown.

Cell population growth (CPG) is the combined outcome of cell proliferation, the increase in cell number resulting from cell division, and cell survival (the suppression of cell death), which prevents the loss of cells [15]. Cancer CPG is crucial to tumor formation and growth, ultimately leading to disease development. Although it has been demonstrated that T2R14 can regulate drug-induced apoptotic cell death in HNSCC cells [14], its role in the growth of oral cancer cells in the absence of treatment, which mimics disease progression, is unknown.

This study aimed to investigate the role of T2R14 in oral cancer CPG. The findings of this study could improve our mechanistic understanding of T2R14’s roles in cancer and provide insight into developing new approaches to advance the treatment of oral cancer.

## 2. Materials and Methods

### 2.1. Chemicals and Reagents

Diphenhydramine hydrochloride (DPH) (O3630) and fetal bovine serum (F1051) were purchased from Sigma-Aldrich (Oakville, ON, Canada); Opti-MEM (minimum essential medium)-reduced serum medium (11058-021), Pen Strep (penicillin streptomycin) (15140-122), D-MEM/F-12 (Dulbecco’s modified eagle medium nutrient mixture F-12 (Ham)) (12400-024), and phosphate-buffered saline (PBS) tablets (18912-014) from Gibco; 0.4% trypan blue (1-450013) from Bio-Rad (Mississauga, ON, Canada); and Lipofectamine 2000 (11668-019) and Fluo-4 NW calcium assay kit (F36206) from LifeScience Technology (Markham, Canada). CRISPR-Cas9 (clustered regularly interspaced short palindromic repeats and CRISPR-associated protein 9) double-nickase plasmids for the control vector (sc-437281) and knocking out *TAS2R14* (sc-410582-NIC) were purchased from Santa Cruz Biotechnology, Inc. ((Mississauga, ON, Canada).

### 2.2. Antibodies

Primary antibodies against proliferating cell nuclear antigen (PCNA) (#13110), mTOR (#2983), and phosphor-mTOR (#5536) were purchased from Cell Signaling Technology (Whitby, ON, Canada). Anti-T2R14 antibody (R61980PA5-102250) was purchased from LifeScience Technology, anti-actin beta (ACTB) (A4700) from Sigma, and goat anti-rabbit IgG (H+L)-HRP (immunoglobulin G (heavy and light chain)-horseradish peroxidase) conjugate (#1721019) and goat anti-mouse IgG (H+L)-HRP conjugate (#1721011) secondary antibodies from Bio-Rad.

### 2.3. Cell Lines and Development of New Cell Lines with TAS2R14 Gene Knockout

Compared with single-cell-derived clones, pooled knockout cells can provide a broader, more representative perspective of the impact of gene downregulation in a heterogeneous population of cancer cells [16]. Thus, we used pooled *TAS2R14* knockout cells in this study. Three OSCC cell lines, including UCSF-OT-1109 (University of California, San Francisco-Oral Tongue-1109) (CRL-3442), CAL27 (CRL-2095) (tongue epithelial cells), and SCC-25 (CRL-1628) (tongue epithelial cells), were purchased from ATCC (American Type Culture Collection, Manassas, USA). UCSF-OT-1109 and CAL27 cells were transfected with CRISPR-Cas9 double-nickase plasmids for the control vector and the vector for knocking out *TAS2R14*, respectively, using Lipofectamine 2000 and Opti-MEM. Then, stable cell lines were developed by using puromycin to kill nontransfected cells, and the pooled cells were used for experiments. The new cell lines were labeled as Mock cells (cells transfected with the control vector (vector alone)) and KO (*TAS2R14* knockout) cells (cells transfected with the *TAS2R14* knockout plasmid). KO was verified by Western blotting for T2R14.

### 2.4. Database Analysis

OncoDB (https://oncodb.org/) (accessed on 6 October 2025) [17,18,19] and Kaplan–Meier Plotter (KM Plotter) (https://kmplot.com/analysis/) (accessed on 6 October 2025) [20] were explored to extract data on *TAS2R14* expression in normal and oral cancer tissues of humans and overall survival information of patients with OSCC or HNSCC, in which OSCC accounts for over 90% of cases [1,2]. The data (graphs) were directly downloaded from the databases, and font size and label locations were adjusted for improved visibility and understanding.

### 2.5. Quantitative Polymerase Chain Reaction

Quantitative polymerase chain reaction (qPCR) was performed using SYBR GreenER™ qPCR SuperMix (Thermo Fisher Scientific (Burlington, ON, Canada), Cat. No. 11762) on an Applied Biosystems QuantStudio™ real-time PCR system. Total RNA was isolated using the RNeasy Mini Kit (QIAGEN (Toronto, ON, Canada), Cat. No. 74104) according to the manufacturer’s instructions. First-strand complementary DNA (cDNA) was synthesized from total RNA using the iScript™ cDNA Synthesis Kit (Bio-Rad Laboratories) following the manufacturer’s protocol. The resulting cDNA was used as a template for qPCR amplification. Gene-specific amplification was carried out using primers for *TAS2R14* (Forward: 5′-TTGGGGCATGCTCTTACAGG-3′; Reverse: 5′-CCCTTGACCCAGTCAATACAGT-3′) and the housekeeping gene glyceraldehyde 3-phosphate dehydrogenase (*GAPDH*) (Forward: 5′-GAAGGTGAAGGTCGGAGTCA-3′; Reverse: 5′ GAAGATGGTGATGGGATTTC-3′). Each reaction was performed in a final volume of 20 µL, containing SYBR GreenER™ qPCR SuperMix, gene-specific primers, and cDNA template. Thermal cycling conditions were as follows: initial denaturation at 95 °C for 120 s, followed by 40 cycles of denaturation at 95 °C for 15 s and annealing/extension at 60 °C for 60 s. A melt-curve analysis was performed from 60 °C to 95 °C at a ramp rate of 0.3 °C/s to verify amplification specificity. Relative mRNA expression levels were calculated using the 2^−ΔΔCt^ method by normalizing to the level of the CAL27 cell line for comparison.

### 2.6. Western Blot

Western blot analysis was performed, as described in the literature [21]. Total cell lysate was generated by lysing cells with NP40 (Nodet P-40 (nonylphenoxypolyethoxyethanol)) (Sigma-Aldrich, I8896) cell lysis buffer containing 1% NP40, 250 mM NaCl, and 50 mM Tris HCl (pH 8.0), with the addition of protease inhibitor cocktail (S59131, Sigma-Aldrich) and phosphatase inhibitor cocktail (D5726, Sigma). Protein concentrations were determined using the Bio-Rad protein assay kit (#5000006), following the manufacturer’s instructions. Equal amounts of protein were separated via SDS-PAGE (sodium dodecyl sulfate-polyacrylamide gel electrophoresis) and transferred onto nitrocellulose membranes (Sigma-Aldrich) at 100 V for 1–2 h. The membranes were blocked with 3% bovine serum albumin or skim milk in PBS containing 0.1% Tween-20 for 1 h at room temperature. After blocking, the membranes were incubated overnight at 4 °C with primary antibodies against the target proteins, followed by incubation with a horseradish peroxidase-conjugated secondary antibody for 1–2 h at room temperature. Protein bands were detected using an enhanced chemiluminescence system (170-5060, Bio-Rad) and imaged on the Bio-Rad ChemiDoc^TM^ MP Imaging System. Densitometric quantification of Western blot bands was performed using ImageJ software (Version: 1.54). To quantify protein band intensities, an area encompassing the entire protein band of a sample with the maximum area of protein band was used for all samples, along with their individual backgrounds, on the same gel. The actual protein band intensity of a sample was calculated by subtracting its background at the upper or lower side of the sample. The relative protein band intensity of a sample was calculated by dividing its corresponding ACTB band intensity.

### 2.7. Intracellular Calcium Mobilization Assay

A Fluo-4 NW calcium assay kit was used to measure the mobilization of intracellular calcium released from the endoplasmic reticulum, as described in our previous publication [22]. OSCC cells were grown in 10 cm dishes till 80–90% confluency. They were split into 96-well clear-bottom, black-walled plates (1 × 10^5^ live cells/well) and were incubated at 37 °C and 5% CO_2_ overnight (16–24 h). Then, the culture medium was removed by aspiration, and cells in each well were incubated with 100 µL Fluo-4 NW dye with probenecid for 30 min at 37 °C and 30 min at room temperature for calcium-mobilization measurement. The working solution of Fluo-4 NW dye was prepared by dissolving the lyophilized dye in 10 mL assay buffer, and probenecid (2.5 mM) was added to block dye leakage from the cytosol by inhibiting the function of specific membrane transporters or channels on the plasma membrane, which can actively pump fluorescent dyes out of the cell. In another 96-well plate with clear bottom and walls, which is called the reagent source plate, 100 µL assay buffer without or with the T2R14 agonist DPH (3 × (1 mM or 2 mM)) was added to each well, where 50 µL of the source (assay buffer without or with DPH) was transferred to the corresponding well in the read plate with cells during calcium measurement. Calcium mobilization was evaluated by measuring relative fluorescence units (RFUs) on a FlexStation 3 multi-mode microplate reader (Molecular Devices, San Jose, USA). The baseline levels of intracellular calcium mobilization were measured for the first 20 s. Then, the reagents in the source plate were added by the built-in 8-channel pipette of FlexStation 3, and RFU readout continued for another 120 s. An absolute (Max–Min) RFU was calculated by subtracting the baseline (minimum (Min)) RFU (before adding the reagent from the source plate) from the maximum (Max) RFU (obtained after adding the reagent). Calcium mobilization (ΔRFU) was calculated by subtracting the absolute RFU in the absence of DPH from that in the presence of DPH.

### 2.8. Cell Viability and CPG Analyses by Counting Cell Numbers

Cells were grown in 10 cm dishes containing complete D-MEM/F-12 medium supplemented with 10% FBS and 1% Pen Strep in an incubator at 37 °C and 5% CO_2_ till 80–90% confluency. Then, the complete D-MEM/F-12 medium was replaced with D-MEM/F-12 medium without FBS (serum starvation) to synchronize cells in the G1 phase of the cell cycle, and the cells were incubated for 48 h before splitting. After serum starvation, the cells were split into 6-well plates, with 0.2 × 10^5^ live cells per well in 3 mL complete D-MEM/F-12 medium. The cells in each well were harvested by collecting live and dead cells, stained with trypan blue by mixing 10 µL cell suspension with 10 µL of 0.4% trypan blue, and counted for cell viability and total cell number on an automated cell counter (Countess 3, LifeScience Technology) after 4 and 6 days of growth, respectively.

### 2.9. Cell Colony Formation Assay

A cell colony formation assay was performed, as described in the literature [21]. Cells were grown in 10 cm dishes containing complete D-MEM/F-12 medium till 80–90% confluency. Then, the complete D-MEM/F-12 medium was replaced with D-MEM/F-12 serum-starvation medium, and the cells were incubated for 48 h before splitting. After serum starvation, the cells were split into 6-well plates, with 2000 live cells per well in 3 mL complete D-MEM/F-12 medium. Cell growth was stopped after 7 days when colonies were visible (≥30 cells per colony). Medium was removed by pipetting, and cells were gently washed twice with 1 mL PBS, then fixed and stained in a mixture of 0.5% crystal violet and 6% glutaraldehyde for at least 2 h. The mixture of crystal violet and glutaraldehyde was removed by pipetting, and cells were washed at least four times with tap water. After the plates containing cell colonies were thoroughly air-dried, the number of colonies in each well was counted using ImageJ software, following image capture.

### 2.10. Statistical Analysis

All data were generated by at least three independent triplicate experiments. Data are represented as means ± standard deviation (*n* ≥ 3). Statistical analyses were performed using GraphPad Prism software version 10.6.0 and Excel 2007. One-way ANOVA (analysis of variance) was used for multiple-group analysis. A two-sample Student’s *t*-test with unequal variances (Welch’s *t*-test) was used for two-group analysis. A value of *p* < 0.05 indicates statistical significance. * *p* < 0.5; ** *p* < 0.01; *** *p* < 0.001.

## 3. Results

### 3.1. T2R14 Is Expressed in Oral Cancer Tissues and Cells

**Figure 1:**
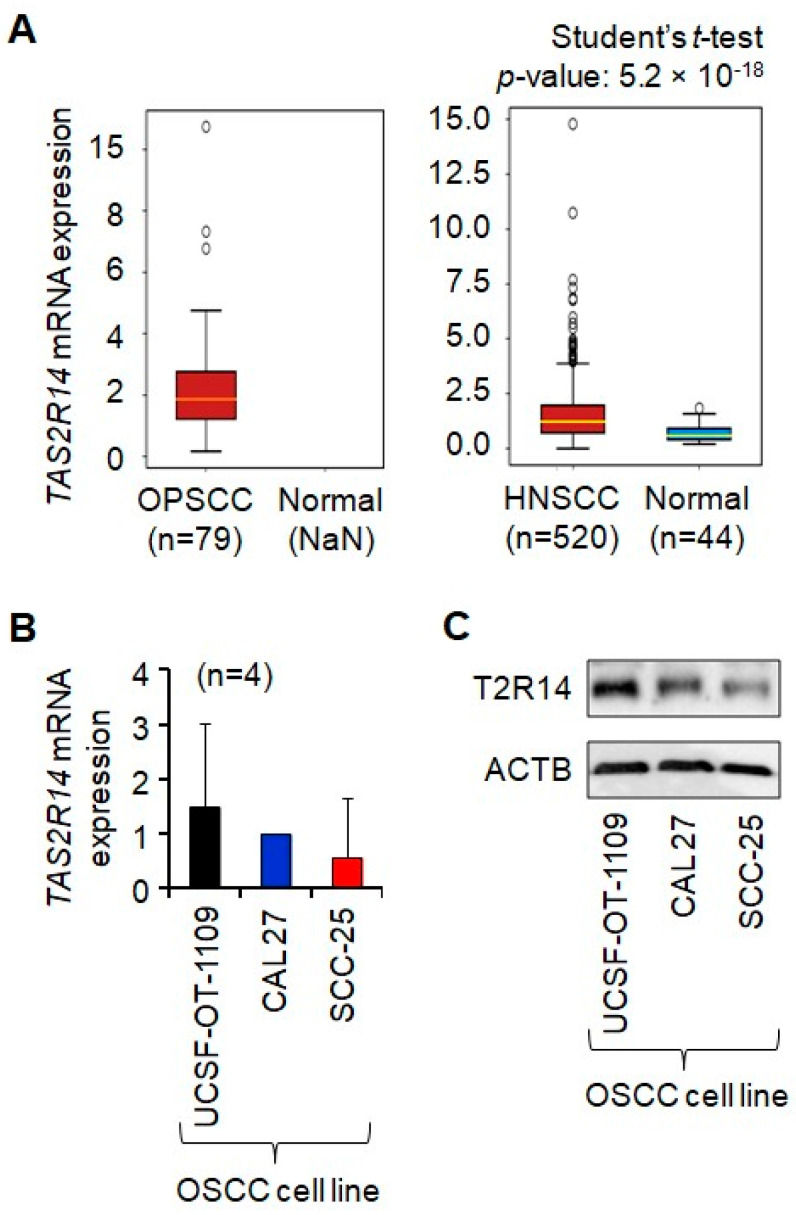
Expression of T2R14 in human oral cancer tissues and cell lines. (**A**) *TAS2R14* mRNA expression in human OPSCC, HNSCC, and normal tissues. Data on mRNA expression were extracted from OncoDB [17,18,19]. (**B**) Expression of steady-state *TAS2R14* mRNA in human OSCC cell lines. mRNA levels were measured by quantitative PCR using *GAPDH* as the housekeeping gene. The data represent four independent experiments (*n* = 4). The mRNA levels of three OSCC cell lines were measured simultaneously in each experiment and normalized to that of CAL27 cells for comparison. No statistical differences in mRNA levels were observed among the three OSCC cell lines (*p* > 0.05). (**C**) Western blot analysis of T2R14 protein expression in human OSCC cell lines. ACTB was used as a loading control. “n” refers to the number of cases/participants. ACTB, actin beta; GAPDH, glyceraldehyde 3-phosphate dehydrogenase; PCR, polymerase chain reaction; T2R14/*TAS2R14*, taste 2 receptor member 14; HNSCC, head and neck squamous cell carcinoma; OPSCC, oropharyngeal squamous cell carcinoma; OSCC, oral squamous cell carcinoma; NaN, not a number (data are missing or unavailable); UCSF-OT-1109, University of California, San Francisco-Oral Tongue-1109, a human OSCC cell line; CAL27, a human OSCC cell line; SCC-25, a human OSCC cell line.

To investigate the role of T2R14 in oral cancer CPG, we first evaluated its expression in oral cancer tissues (in vivo) and in oral cancer cells (in vitro). mRNA analysis of the OncoDB database (data generated by other researchers) shows that *TAS2R14* mRNA is expressed in human oropharyngeal squamous cell carcinoma (OPSCC), a type of OSCC, and HNSCC, in which OSCC accounts for over 90% of cases, as well as in normal tissues [17,18,19] (Figure 1A). Furthermore, the steady-state mRNA and protein of T2R14 were expressed in OSCC cell lines: UCSF-OT-1109, CAL27, and SCC-25 (Figure 1B,C). No statistical differences in *TAS2R14* mRNA levels were observed among the three OSCC cell lines (*p* > 0.05). SCC-25 showed the same trend, expressing lower levels of T2R14 mRNA and protein than the other two cell lines. *TAS2R14* mRNA levels are significantly higher in HNSCC tumors than in normal tissues (Figure 1A, right panel); however, *TAS2R14* mRNA data from the normal tissues (NaN refers to data missing or unavailable) are unavailable in the cohort with OPSCC tumors (a type of oral cancer tumor) versus normal tissues (Figure 1A, left panel). Differences in T2R14 mRNA and protein levels among three OSCC cell lines from different backgrounds reflect heterogeneity, a common feature of cancer. These data support the expression of T2R14 in oral cancer tissues and tongue cancer cell lines.

### 3.2. T2R14 Downregulation Decreased Its Activation in Oral Cancer Cells

**Figure 2:**
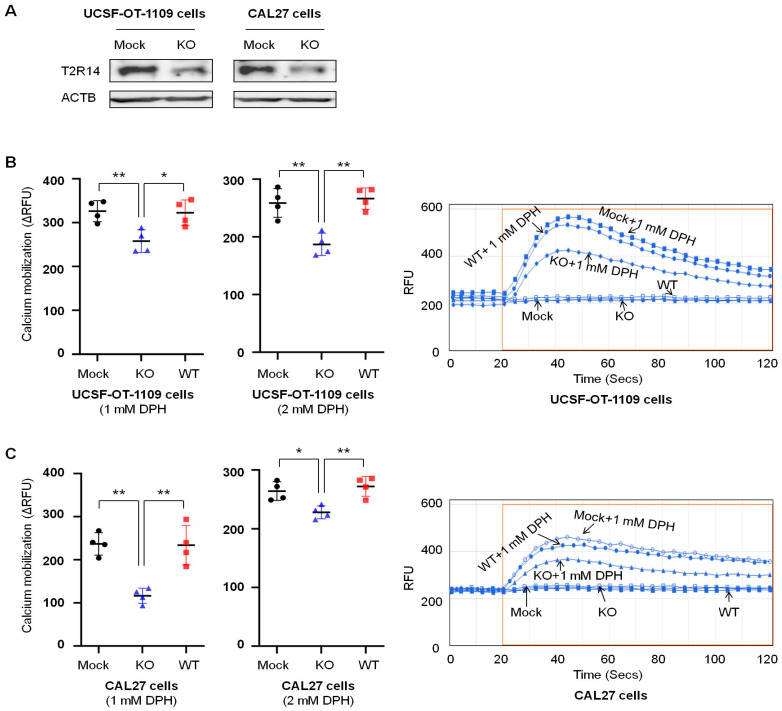
Effect of T2R14 downregulation on calcium mobilization in oral cancer cells. Calcium activation was evaluated by measuring intracellular calcium mobilization using the agonist DPH. (**A**) Western blot showing *TAS2R14* knockout. (**B**) Effect of *TAS2R14* knockout on calcium mobilization in the presence of the agonist DPH at 1 mM and 2 mM in UCSF-OT-1109 cells. A representative histogram image of calcium mobilization with and without 1 mM DPH is shown on the right. Results represent four independent triplicate experiments. Each dot represents the average value of three replicates. (**C**) Effect of *TAS2R14* knockout on calcium mobilization in the presence of its agonist DPH at 1 mM and 2 mM in CAL27 cells. A representative histogram image of calcium mobilization with and without 1 mM DPH is shown on the right. Results represent four independent triplicate experiments. Each dot represents the average value of three replicates. DPH, diphenhydramine hydrochloride; RFU, relative fluorescent unit; ΔRFU, RFU increase (maximum RFU-minimal RFU) in the presence of DPH minus RFU increase (maximum RFU-minimal RFU) in the absence of DPH; Mock, stable cell line post-transfection with vector alone; KO, stable cell line post-transfection with the vector for *TAS2R14* knockout; WT, original cell line with endogenous T2R14 and without transfection; Secs, seconds. * *p* < 0.05; ** *p* < 0.01.

One well-known feature of T2R14 is that it mobilizes (increases) intracellular calcium upon activation. To evaluate the impact of T2R14 in oral cancer cell lines, we knocked out *TAS2R14* using the CRISPR-Cas9 pooled technique and compared the results from the KO cells to those from the wild-type (WT) cells (without transfection) and Mock cells. Our previous study showed that the T2R agonist DPH can activate T2R14 with an EC50 (half-maximal effective concentration) of 0.5–0.7 mM and an Emax (maximal effective concentration) of 1–2 mM, depending on the cell types [23,24]. Therefore, we used DPH as a T2R14 agonist at 0.5 mM, 1 mM, and 2 mM to evaluate the effect of *TAS2R14* knockout on calcium mobilization. *TAS2R14* knockout (Figure 2A) significantly inhibited calcium mobilization by 20–30% in the presence of the T2R14 agonist DPH at 1 mM or 2 mM in UCSF-OT-1109 cells (Figure 2B) and by about 50% with 1 mM DPH and 10–20% with 2 mM DPH in CAL27 cells (Figure 2C). Variable low RFU signals were observed with 0.5 mM DPH. The calcium mobilization data demonstrated that *TAS2R14* knockout decreased DPH-mediated T2R14 activation and confirmed T2R14 downregulation by the CRISPR-Cas9 pooled approach used in this study. Because no significant differences in calcium mobilization were observed in WT and Mock cells from both UCSF-OT-1109 and CAL27 cell lines, we used Mock cells for the following experiments.

### 3.3. T2R14 Downregulation Increased Oral Cancer CPG

**Figure 3:**
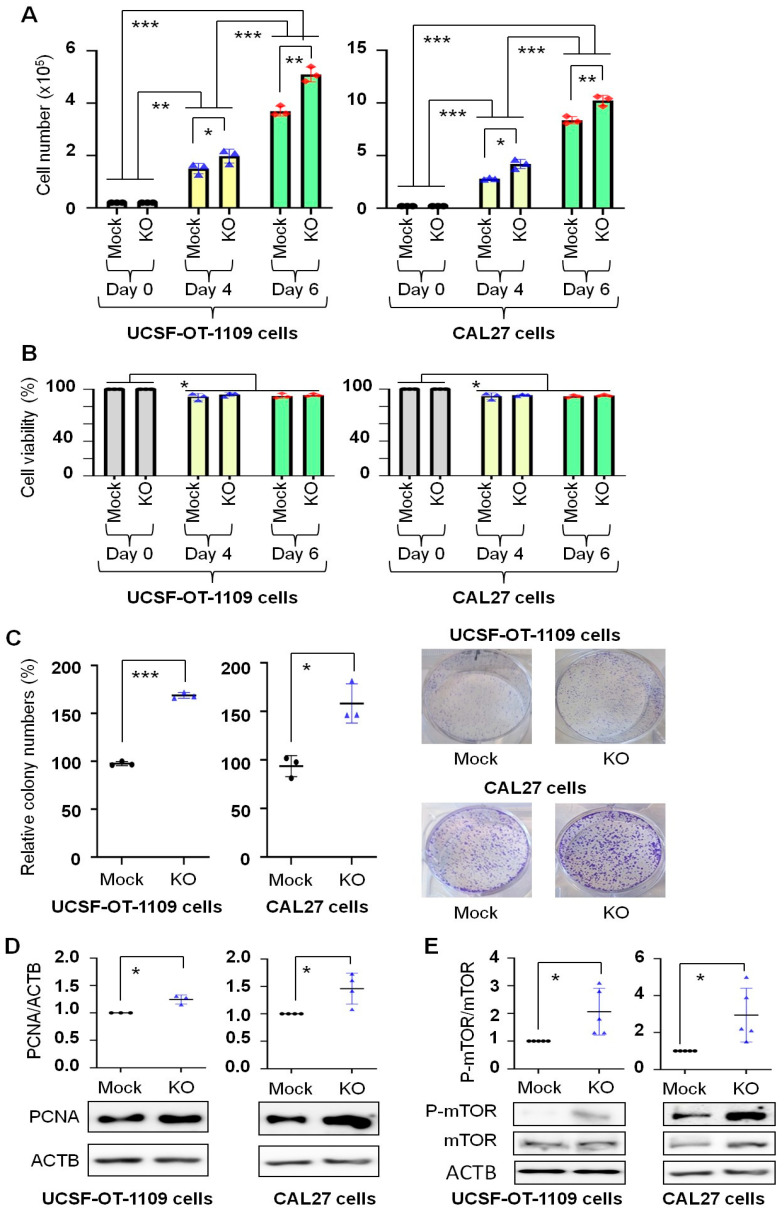
Effect of T2R14 downregulation on oral cancer cell population growth. Effect of *TAS2R14* knockout on cell number change (**A**) and cell viability (**B**) following 6-day cell growth, after the same number of live cells (0.2 × 10^5^) was placed in each well of 6-well plates. Results represent three independent triplicate experiments. Each dot represents the average value of three replicates. (**C**) Effect of *TAS2R14* knockout on colony formation. In each well of 6-well plates, 2,000 live cells were added in complete medium, and cell colonies were counted following staining with crystal violet on Day 7, as described in the Materials and Methods section. Results represent three independent triplicate experiments. Each dot represents the average value of three replicates. Representative images of colonies in 6-well plates are shown on the right. (**D**) Western blot analysis of the effect of *TAS2R14* knockout on the expression of the proliferation marker PCNA. Quantification results represent three (for UCSF-OT-1109 cells) or four (for CAL27 cells) independent experiments. Representative images of protein bands are shown below the quantification graph. ACTB was used as a loading control. (**E**) Western blot analysis of the effect of *TAS2R14* knockout on the expression of mTOR and P-mTOR. Quantification results represent five independent experiments. Representative images of protein bands are shown below the quantification graph. *TAS2R14,* taste 2 receptor member 14; Mock, stable cell line post-transfection with vector alone; KO, stable cell line post-transfection with the vector for *TAS2R14* knockout; PCNA, proliferating cell nuclear antigen; ACTB, actin beta; mTOR, mechanistic target of rapamycin; P-mTOR, phosphor-mTOR (Ser2448); UCSF-OT-1109, University of California, San Francisco-Oral Tongue-1109, a human OSCC cell line; CAL27, a human OSCC cell line. * *p* < 0.05; ** *p* < 0.01; *** *p* < 0.001.

CPG is the sum of cell proliferation and cell viability, a net balance between cell number increase from proliferation and cell loss due to cell death. Therefore, cell number change and cell viability were assessed after the cells were incubated in growth medium for a long time (up to 6 days). Figure 3A shows that in cell lines developed from both UCSF-OT-1109 and CAL27, cell numbers increased with incubation time in Mock and KO cells, and significantly more cells were observed in KO cells than in Mock cells at Day 4 and Day 6. Cell viability decreased by less than 10% on Day 4 or Day 6 compared to that on Day 0; however, no differences were observed between Mock and KO cells on Day 4 or Day 6 (Figure 3B). These phenomena support that the higher cell numbers observed on Day 4 and Day 6 in KO cells than in Mock cells result from increased cell proliferation. This conclusion cannot be made if cell viability were lower in Mock cells than in KO cells. The colony formation assay measures a single cell’s ability to survive the growing microenvironment and form a population (visible colony), a net outcome of long-term cell proliferation and survival (resistance to cell death) [25]. Consistent with the data on cell number change (Figure 3A), more colonies were formed from KO cells than from Mock cells after the same number of cells were grown for 7 days, from both UCSF-OT-1109 and CAL27 cells (Figure 3C). These observations indicate that T2R14 downregulation increased oral cancer CPG, with proliferation being the primary contributing factor. Because PCNA is a proliferation marker of oral cancer [26,27], we evaluated its expression following *TAS2R14* knockout. Figure 3D shows that PCNA was significantly upregulated by *TAS2R14* knockout in both UCSF-OT-1109 and CAL27 cells, further supporting the fact that T2R14 downregulation increased oral cancer cell proliferation. Activation of the mTOR complex 1 (mTORC1) pathway is well-known to promote cell proliferation and growth [28,29]. Figure 3E shows that *TAS2R14* knockout significantly increased mTORC1 activation (phosphorylation of mTOR at Ser2448) in both UCSF-OT-1109 and CAL27 cells. The data suggest at least a plausible mechanism for increased oral cancer cell proliferation induced by T2R14 downregulation.

### 3.4. T2R14 Expression Is Associated with the Overall Survival of Patients with Oral Cancer

**Figure 4:**
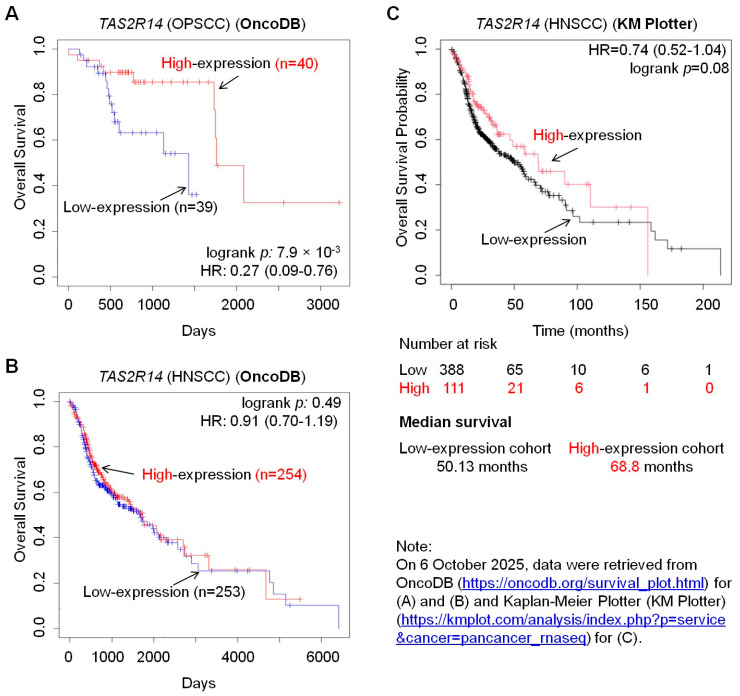
Database analyses of patient survival. Data on the overall survival of patients with OPSCC (**A**) or HNSCC (**B**) with low and high expression of *TAS2R14*. Data were extracted from OncoDB (https://oncodb.org/) [17,18,19]. (**C**) Data on the overall survival probability of patients with HNSCC with low and high expression of *TAS2R14*. Data were extracted from the Kaplan–Meier Plotter (KM Plotter) (https://kmplot.com/analysis/) [20]. The value of “n” refers to the number of cases/participants. *TAS2R14*, taste 2 receptor member 14; HR, hazard ratio; HNSCC, head and neck squamous cell carcinoma; OPSCC, oropharyngeal squamous cell carcinoma.

To investigate the clinical implications of T2R14-mediated oral cancer CPG, we searched online cancer databases. According to OncoDB [17,18,19], patients with OPSCC who have high *TAS2R14* expression have significantly higher overall survival than those with low *TAS2R14* expression (Figure 4A). The same trend is also observed in patients with HNSCC based on OncoDB (Figure 4B) and Kaplan–Meier Plotter (KM Plotter) (https://kmplot.com/analysis/ (accessed on 21 August 2025)) [20] (Figure 4C). These observations align with the results presented above: *TAS2R14* knockout increased oral cancer CPG. Low levels of T2R14 increase oral cancer CPG, promoting oral cancer disease progression and decreasing patient survival; in contrast, higher levels of T2R14 might inhibit oral cancer CPG and disease progression, thereby increasing patient survival.

## 4. Discussion

Oral cancer is a malignancy significantly affecting close to 400,000 individuals and leading to about 200,000 deaths annually [3]. However, many of its mechanisms remain unexplored. The 5-year overall survival rate for OSCC remains at 50–60% [30], underscoring the need to improve its diagnosis and treatment. GPCRs play crucial roles in cancer and are important therapeutic targets for the disease [31]. T2R14 is a GPCR that has attracted increasing attention in cancer research over the past few years. However, its role in cell proliferation and CPG of oral cancer cells remains unknown. This study demonstrated that *TAS2R14* knockout significantly increased proliferation and CPG of oral cancer cells, at least by activating the mTORC1 pathway. Cancer cells need to accumulate to an adequate number for disease progression, which requires increased CPG. The findings of this study indicate that low levels of T2R14 increase oral cancer CPG, thereby promoting oral cancer progression and adversely affecting patients; in contrast, high levels of T2R14 inhibit the growth, thereby suppressing disease progression and benefiting patients. This notion is supported by clinical data showing that high *TAS2R14* expression is associated with better overall survival in patients with OPSCC or HNSCC (Figure 4). Therefore, T2R14 might play a tumor-suppressor-like role in oral cancer progression.

The effect of T2R14 on cell proliferation has been implicated in several previous studies. Our previous study demonstrated that activation of T2R14 by the agonists quinine and apigenin can inhibit breast cancer cell proliferation [32]. Lidocaine-induced activation of T2R14 inhibits apoptotic cell death and proliferation in OSCC cells [14]. A recent study reported that T2R14 can drive pro-ferroptotic cell death and inhibit the proliferation of thyroid cancer cells [33]. However, the role of T2R14 in oral cancer cell proliferation is unknown. The findings of this study timely fill the gap in the field. This study demonstrated that T2R14 downregulation increased oral cancer cell proliferation, consistent with the previous findings that T1R14 inhibits cell proliferation in various cancer cell types, as mentioned above. We showed that T2R14 downregulation increased PCNA expression, consistent with the lower overall survival in patients with OSCC or HNSCC (Figure 4) and reports that PCNA index can be used to evaluate proliferation and aggressiveness in dysplasia and different grades of OSCC [34]. Activation of mTORC1 signaling is a well-known mechanism of increased proliferation and growth of cancer cells [29], including oral cancer cells [35]. This study showed that T2R14 downregulation promoted oral cancer CPG, at least in part, by activating mTORC1.

Figure 1A shows that *TAS2R14* mRNA levels are significantly higher in HNSCC tumors than in normal tissues. This observation does not align with the data in Figure 4 or our conclusion of a tumor-suppressor role for T2R14. This discrepancy warrants further studies and may be due to differences in *TAS2R14* mRNA transcription, genetic variants, and/or its stability in HNSCC cancer cells versus normal cells. Our recent in silico analysis using RNAsnp suggests that single-nucleotide variants in T2Rs can differentially influence mRNA secondary structure depending on genetic context [36].

Previous studies from our group and others have focused on T2R14 activation via its agonists. In Figure 3, we show that in the absence of a known T2R14 agonist, T2R14 downregulation increased CPG. This phenomenon might be independent of T2R14 activation or related to reduced T2R14 activation in the presence of endogenous agonists. The detailed mechanisms warrant further research. Our findings are consistent with previous studies showing that T2R14 activation is involved in cell death in HNSCC cells [6,37], as cell death reduces cell numbers and thereby CPG. These observations support the hypothesis that T2R14 might suppress CPG of cancer cells.

This study is novel as it is the first study to investigate the role of T2R14 in proliferation and CPG of oral cancer cells. Furthermore, this study demonstrated that T2R14 downregulation increased CPG of oral cancer cells even in the absence of a known T2R14 agonist. These findings open a new avenue of research to explore the functions of T2R14 and other bitter taste receptors independent of receptor activation or in the absence of known agonists or activators. However, this study has some limitations. We evaluated the roles of the proliferation marker PCNA and mTORC1 signaling in T2R14-mediated oral cancer CPG; however, we did not investigate other proliferation markers and cell signaling pathways, and this will require further investigation. Oral cancer progression involves tumor growth; however, the role of T2R14 in oral tumor development and growth has not been assessed.

In summary, this study, for the first time, demonstrated that T2R14 downregulation increased oral cancer CPG by upregulating the proliferation marker PCNA and mTORC1 signaling. The observations are consistent with clinical data from online databases, which indicate that higher *TAS2R14* expression is associated with better survival in patients with OSCC or HNSCC. These findings support that T2R14 is a potential tumor suppressor in oral cancer. However, future studies should further explore the mechanisms underlying T2R14-regulated oral cancer CPG, such as evaluating the roles of various proliferation markers and the upstream and downstream signaling of the mTOR pathway and other signaling pathways, as well as the impact of T2R14 on tumor initiation and growth in vivo. The findings of this study update our knowledge and research approaches in CPG in cancer biology; they could provide insight into developing strategies to improve treatment for oral cancer and other cancers by targeting T2R14. For example, approaches to increase T2R14 expression or reduce its intracellular degradation to improve oral cancer treatment can be developed. Since T2R14 activation can help induce apoptosis [14], activating T2R14 with its agonists is another approach to target it in oral cancer treatment.

## Data Availability

Data are available upon reasonable request from the corresponding authors.

## Abbreviations

The following abbreviations are used in this manuscript:

ACTB	Actin beta
CAL27	A human squamous cell carcinoma cell line derived from a tumor in the tongue tissue
CPG	Cell population growth
DPH	Diphenhydramine hydrochloride
GAPDH	Glyceraldehyde 3-phosphate dehydrogenase
GPCR	G-protein-coupled receptor
HPV	human papilloma virus
HNSCC	Head and neck squamous cell carcinoma
KO	*TAS2R14* knockout
mTOR	Mechanistic target of rapamycin
mTORC1	Mechanistic target of rapamycin complex 1
OPSCC	Human oropharyngeal squamous cell carcinoma
OSCC	Oral squamous cell carcinoma
PBS	Phosphate-buffered saline
PCNA	Proliferating cell nuclear antigen
qPCR	Quantitative polymerase chain reaction
RFU	Relative fluorescence unit
SCC-25	A human squamous cell carcinoma cell line derived from a tumor in the tongue tissue
T2R14/*TAS2R14*	Taste 2 receptor member 14 (also called bitter taste receptor 14)
UCSF-OT-1109	University of California, San Francisco-Oral Tongue-1109
WT	Wild-type
